# Report of Surgical Cases at the Buffalo General Hospital, with Clinical Remarks

**Published:** 1875-02

**Authors:** Julius F. Miner


ART. III.—*Report of Surgical Cases at the Buffalo General Hospital, with Clinical Remarks by Professor Julius F. Miner.* By BERNARD BARTOW, M. D., Resident Physician, and W. W. MINER, M. D.

AMPUTATION OF FINGERS—HISTORY.—H. H., aet. 14, German, laborer. Three days previous to entering hospital, (Nov. 6, 1874,) the patient had his right hand caught between the rollers of a machine used for crushing bones. The skin upon the back of hand was torn off in one large flap, exposing the muscles; the phalanges of all the fingers were comminuted, and in places protruded. The wounds were filled with bone dust, acids, etc. The thumb escaped injury. Gangrene of the fingers and flap ensued, though strenuous efforts had been made to save them. It seemed probable that the whole hand might suffer in like manner.

Clinical Remarks.—The condition here is one which unmistakably demands amputation. I find it best to amputate at the metacarpo-phalangeal articulations. In doing this you notice that a portion of the extremities of the metacarpal bones are left uncovered by integument on their dorsal aspect, where it was destroyed by the sloughing process. Accordingly I do not attempt to close the wound with stitches, but direct the application of warm water dressings, soon to be followed by a poultice dressing which will envelop the whole wound. Ten or twenty years ago I should not have done thus. In these cases of injury to the hand the important thing is to save, not to cut off. If parts are unmistakably not to be saved, amputate, otherwise wait till you find what is dead and what is alive. The hand cannot be supplied with an artificial substitute that is satisfactory to any where near the degree that an artificial leg may be. Cut off nothing that can be retained in the hand. Examples and cases that you observe, alone teach you what can be retained or what it is best to do in these cases. This is a real test of the ability of the surgeon. Any one can perform the mechanical part of operations. To decide whether removal is necessary, and if so, how and where it shall be done, will test the ability more than the performing of the operation in cases of hand surgery at least.

Case—Syphilitic Nodes and Erosion—History.—The patient with the nodes says she has been sick about one year. She has no knowledge of having had primary syphilitic lesion; has never noticed an eruption upon the body or limbs. She has had sore throat, loss of hair, headache and nocturnal pains; in addition, she has a large circular unhealthy ulcer over the right tibia. There is also a node upon the opposite tibia which presented the signs of having suppurated, being red, painful and fluctuating; a free incision, however, proved the appearances deceptive, there being instead of fluid an elastic gelatinous material, which subsequently suppurated.

The nodes and ulcer made their appearance about six weeks since.

The patient was given potass. iod. gr. xx, four times daily; also ferri. et potass. tart. gr. x. ter in die. directed to the condition of anæmia.

The patient with the phagadenic ulcer upon the leg, entered the hospital in a broken down condition, having in addition to her syphilitic trouble, erysipelas. The ulcer at first the size of a child's hand, became gangrenous, dissecting the muscles, and nearly girdling the limb, for a space of eight inches, before the sloughing process was controlled.

Of the various applications used, none seemed as efficient as packing the ulcer with lint, soaked in a solution of carbolic acid, containing one part acid in four parts water.

The patient had been taking fifty grains potass iod. daily for a week, when the amount was increased to one hundred grains daily with marked benefit. The potassio-tartrate of iron was also given in respect to its anti-phagadenic properties.

Clinical Remarks.—You have observed in one of the wards a patient presenting a marked condition of anemia and prostration, while upon both her arms are present numerous bunches, of considerable size, which give quite a look of deformity to each forearm. I have never asked the patient the cause of her troubles. I know perhaps better myself than the patient, that within five or ten years since there was present an infecting chancre, followed by a sore throat, with mucous patches in the fauces, an eruption on face or more extensive, with possibly still other troubles. You can sometimes distinguish the disease by its characteristic odor, and recognizing this, you can infer its whole history. The primary symptoms always appear within the three months succeeding infection; the secondary within the first year, and afterwards the tertiary. The system shows the first effects of infection in the secondary period. You have also seen to-day a leg that looks as if it were going to drop off from syphilitic erosion, which has destroyed the soft structures more than half way through the leg. Both cases are due to one source. The treatment of each is with iodide of potassium in ten or twenty grain doses; twenty grains would not be too much, and from that up to sixty grains may be given. I not infrequently find physicians giving one or one and a half grains, three times a day, and wondering why their patients do not get well. A ten grain dose is a common one, and patients take one hundred grains thrice daily with no injurious effects. Give iodide of potassium in adequate doses, increase your dose until it is adequate. In the latter stages of syphilis it is almost specific. Mercury may be added to this if there is any delay in desired effects. Mercury in secondary syphilis is more of a specific than any other medicine in the materia medica. Iodine or iodide of potassium in tertiary syphilis is as much a specific as quinine is in intermittent fever; with mercury and iodine we can most signally control syphilitic disease.

Case—Dislocation of the Shoulder—History.—The patient states that three days since, while walking he slipped upon some ice, and in the endeavor to save himself reached for a barrel near by. His arm being extended laterally struck upon the top of the barrel breaking his fall, but throwing the head of the humerus out of its socket and into the axilla.

Clinical Remarks.—The young man here has had this condition of his arm for several days back. He has a degree of immobility of the shoulder joint; his elbow is held in a position unnaturally removed outwards from his side; the contour of his shoulder is imperfect. What do these symptoms plainly indicate? It is dislocation of the humerus downwards. Difficulty in recognizing these symptoms occasionally occurs, and if there is failure in this respect, bad results follow. If neglected for some time the arm is apt to become more or less paralyzed and atrophied. I have in mind a case in which I affected a reduction six or eight weeks after dislocation, and there remains a half paralyzed condition which disables the limb. Complete anæsthesia is necessary before attempting reduction, incomplete anæsthesia is worse than none. There has not been much pain present in this case, though that is frequent from the pressure of the head of the bone upon the brachial plexus of nerves. Return of the dislocated surfaces takes place with a distinct sound that all could hear. You can see that motion of the joint is perfect now. The arm may be folded to the breast and bound to the side with a bandage. This after treatment need be employed but for a short time.

